# On Bilious Dyscrasy (Icterus) with Acute Yellow Atrophy of the Liver

**Published:** 1846-10

**Authors:** 


### ART. II.

*Die Gallige Dyscrasie (Icterus) mit acuter gelber Atrophie der Leber.* Von Dr. P. J. HORACZEK, practischen Arze in Wien, &c.—*Wien,* 1844.

*On Bilious Dyscrasy (Icterus) with Acute Yellow Atrophy of the Liver.* By Dr. P. J. HORACZEK.—*Vienna,* 1844. 8vo, pp. 278.

THIS is an enlarged edition of a work that has been received with considerable favour; and in its present altered and amended form, is intended by its author, to serve as an introduction to the subject of biliary disease in general.

Dr. Horaczek points out the confusion and error that have been introduced into this important department of pathological inquiry by preceding writers, who have either confounded diseases essentially different under the same appellation, or have applied different names to diseases closely allied, if not actually identical.

He objects to the names icterus and jaundice as expressive of a symptom merely, and proposes to substitute for them the terms "bilious dyscrasy," "cholosis," or "cholomæa," as significant of the peculiar dyscrasy of the blood, and of its saturation with more or fewer of the elements of the bile, that constitute the essence of this group of diseases.

The disease he subdivides as follows: 1, primary, or idiopathic, or protopathic cholosis; 2, secondary, or deuteropathic, or symptomatic cholosis; 3, polycholia.

*Primary, or idiopathic bilious dyscrasy,* or cholosis, which is the subject of the work before us, immediately consists, the author tells us, of a diseased composition of the blood, and a contemporaneous disturbance or total suspension of the secreting functions of the liver; both being the result of a disaccordance or derangement of the due vital relations between the nervous and vascular systems. Various anatomical and physiological changes are constant attendants on these primary diseased conditions, of which the most characteristic is a diminution in volume and alteration in texture of the liver, which, under the name of *acute yellow atrophy,* he has made part of his nosological definition.

To primary bilious dyscrasy belong the greater number of rapidly fatal cases distinguished as jaundice, as well as many known under the names hepatitis, bilious fever, cephalocholosis, &c.

*Secondary bilious dyscrasy* originates in various structural changes of the liver itself or of the neighbouring organs, in consequence of which, bile is again taken up into the circulation after its formation, and the functions of the liver are mechanically impeded.

*Polycholia* consists in an inordinate formation of the constituent materials of the bile, and their accumulation in the blood, concurrently with undiminished or even increased functional activity of the liver.

The earliest and most important change characteristic of primary bilious dyscrasy is that which takes place in the blood. In consequence of a deficiency of fibrine, icteric blood coagulates imperfectly. The coagulum breaks down easily, and is of a dark yellowish brown or dirty red colour, little altered by contact with the atmospheric air. As far as can be ascertained by microscopic investigations, the blood-corpuscles are diminished in number, and swollen. They also vary from each other in shape, and are less clearly defined in figure than natural. The serum is opaque and viscid, and it, as well as the buffy coat, when the latter does occur, is deeply yellow from a large admixture of the colouring principles of the bile. The other biliary constituents are not found in the blood in cases of purely idiopathic cholosis; though the peculiar atomic combinations from which the liver prepares the bile, as well as the biliary acids, sometimes are. The peculiar atrophy of the liver involves shrinking to a half or even a fourth of its usual size, with wrinkling of its peritoneal coat. Its parenchyma is soft, flaccid, bloodless, and inelastic, of a greenish or dirty orange colour; and deprived of its peculiar granular structure, so that its different constituents can no longer be distinguished from each other. The gall-bladder is generally found contracted, and contains a colourless, or gray, or greenish slimy bile; its ducts are contracted and empty.

The spleen is in almost every case enlarged to twice or thrice its usual size, and easily lacerable or even reduced to a dark red pulp.

In severe and fatal cases the brain is found to have undergone serous infiltration, and great softening, especially round the central white parts. Yellow serum is found poured out as well over the convolutions of the brain and base of the skull as in the ventricles.

Anemia and relaxation of tissue or softening are found to be conditions common to the muscular, nervous, vascular, and glandular structures.

The mucous membranes in general, and the gastric mucous membrane in particular, are frequently softened. The mucous coat of the stomach, especially at the greater curvature, is found reduced to a dirty gray or greenish pulp. This change sometimes extends to the œsophagus, but more frequently to the duodenum; it is most frequently met with in the case of children.

Besides the foregoing, which Dr. Horaczek looks upon as constant and characteristic results of primary bilious dyscrasy, there are others of a casual or occasional character, of which the most important are congestions of the lungs, and passive inflammations of those organs or of the pleura or peritoneum; the products of which indicate the cacoplastic and diseased character of the blood.

The formation of coagula and limited inflammations in the portal or neighbouring veins, with consecutive purulent deposits in the liver, lungs, or other organs, are rare but very serious attendants on primary cholosis.

After having enumerated the symptoms which usually mark the onset of the disease, such as gastric and enteric disturbance, languor, lassitude, incapability of, or disinclination to, mental or bodily exertion, irregular chills and heats, loss of rest, depression of spirits, irritability of temper, &c.; the author enters at some length upon the consideration of the attendant febrile disturbance and characteristic yellowness.

The fever which seldom goes beyond mere irritative reaction, has generally an irregularly remittent character; the exacerbations assuming a regular quotidian or tertian type, only in situations favorable to the development of intermittent fever, or in the case of persons predisposed to neuroses of the ganglionic nerves. In many instances it disappears altogether after a few days. In the case of strong young persons, or those characterized by great vascular or nervous irritability, who have been subjected to the action of strong exciting causes, the disease often sets in violently with spasms or convulsions of an epileptic, tetanic, or hydrophobic character, or even with apoplectic symptoms; the attendant fever then wears an inflammatory aspect, with severe pain in the head, region of the liver, or abdomen, general turgescence, obstinate vomiting, red tongue, injected eyes, diminished secretions, heat, thirst, increased sensibility to light and sound, and full, hard, frequent, contracted pulse. After some days this high febrile action ceases, leaving behind a weak, soft, slow, and unequal pulse, or it may continue in a moderate form during the greater part of the illness, and terminate only with the general decline of the symptoms, or under imperfect critical efforts, manifested by general perspiration, deposits in the urine, bilious stools, or hemorrhage. The characteristic and never-failing discoloration which attends cholosis, extends over the surface sometimes in the course of a few hours, and also stains every organ and tissue in the body, except the substance of the brain and nerves, and the enamel of the teeth. It is obviously the result of vicarious efforts to get rid of the biliary elements accumulated in the blood, and varies from a clear or dark yellow to a green bronze, and even reddish hue, according to race, occupation, and the presence of other cachectic conditions. Thus, in cases of chlorotic complication it is of a grayish; in scorbutus, of a greenish or dark yellow tint. Other modifications of which it is susceptible, which have led to the adoption of distinctive appellations, such as icterus variegatus, ict. partialis, ict. dimidiatus, &c., generally belong to the secondary forms of cholosis, and depend on the coexistence of other dyscrasies or on organic disease.

The presence of the biliary colouring materials in the urine is shown by its characteristic saffron yellow colour when seen in thin layers, and also by its power of staining other objects, as well as by the effects of nitric and hydrochloric acids. If the freshly passed urine be carefully poured on a sufficient quantity of nitric acid, and the two be gradually mixed together, the red or yellow colour of the urine will pass into green, blue, and violet. If the colouring material in the urine be small in quantity, the change to green is succeeded by a restoration of the yellow colour, without the intervention of any intermediate tint. The yellow or brown colour of the urine, is also changed to green by hydrochloric acid, but less certainly and obviously; the smallest colouring admixture of the urine may also be detected by using a good achromatic microscope.

The other constituents of the bile, such as biliary resin, bilin, cholesterin, &c., are also present in icteric urine. The cholesterin is in some cases deposited, and may be detected under the microscope as regular prismatic crystals, or more frequently as an amorphous mass. Icteric urine, when voided, throws up a permanent froth, has a saponaceous feel, gives out a peculiar sickly sweetish smell, and deposits on the vessel a fatty opalescent scum.

When the disease is at its height the urine is alkaline, and disposed to putrefy; but as the symptoms decline it becomes acid soon after emission. The urea is also generally diminished, as well as the fixed salts and earthy phosphates. The specific gravity varies a good deal; it is often normal, but still more frequently falls to 1010-1008. These changes Dr. Horaczek looks upon as resulting from the diminished energy of the vascular system, and consequent imperfection of the respiratory functions, the effect of which is, that a smaller quantity of oxygen is brought into contact with the blood, and the decarbonization of its normal and abnormal constituents is diminished; and hence, as in the case of the amphibia, the formation of urea is lessened, and that of uric acid is increased.

The taste and reaction under nitric acid show that the biliary constituents are present in some of the other secretions, amongst which may be mentioned the perspiration which tinges linen—the serum, whether of the blood or that poured out by blisters, or from œdematous parts—the various mucous discharges, and those from abscesses and ulcers.

The paucity of observations does not allow us to decide whether the tears, saliva, semen, and milk, undergo similar changes. But the bitter taste of the milk observed by some, and the refusal of the breast by the infant, show that it must have sustained some qualitative change.

The characteristic pain of the liver, which sets in at the commencement of the disease, and accompanies it throughout, is of a nervous character, and is generally referred to a circumscribed spot over the left lobe. In slight cases it amounts only to a feeling of tension and oppression, with occasional fugitive stitches; aggravated by pressure so as to compel the patient to make a forced respiration. In bad cases it becomes very severe. It goes hand in hand with the loss of function and decrease in size of the organ.

The increase in volume of the spleen, which keeps pace with the atrophy of the liver, is evidenced by feelings of tenderness and oppression in the left hypochondrium, which sometimes increase to severe pain obstructing respiration.

There is also a remarkable pain in the eighth or ninth dorsal vertebra; as well as pains in the limbs and joints of a rheumatic character, which alternate with a feeling of stiffness, and a consciousness of stupor, or total loss of sensation in the parts. As these painful affections are witnessed in cases where there has been violent vomiting; Dr. Horaczek thinks they may be attributed to the mechanical agitation of the body. Embarrassed respiration and constriction of the chest, which are increased at night, or on making any sudden movement, and sometimes induce fits of palpitation, he attributes to the diseased condition of the blood. Disease of the heart or lungs inducing similar symptoms may be detected by percussion and auscultation. The advanced stages and severer forms of the disease are marked by cephalic and nervous symptoms, which set in sometimes quite suddenly and unexpectedly. To restlessness, sleeplessness, high delirium, repeated vomiting, and tonic or clonic spasms, succeed apathy, diminished sensibility to external impressions, low muttering delirium, with rapid, feeble, unequal pulse, gradually increasing, and at length profound coma, paralytic affections, total unconsciousness, involuntary evacuations, cold viscid sweats, and death. This modification of the disease has been named nervous jaundice, or cephalocholosis. Sometimes the disease runs on to regular typhus, or it ends in dysentery, or the powers sink under coffee-ground vomiting and purging, indicative of passive hyperemia, and softening of the mucous lining of the intestinal canal.

Of combinations and complications, besides those just noticed the most serious is that with inflammation of the abdominal veins, which the author thinks may be attributed to the rapid shrinking of the liver inducing retarded circulation and partial coagulation of the blood. The coagulum so formed undergoes a purulent decomposition, and induces inflammation of the inclosing vessels, attended by the usual symptoms. One very important remark, especially in a practical point of view, is, that genuine healthy inflammation is incompatible with cholosis; though inflammatory congestion with softening of the mucous and serous membranes, and passive inflammations of the lungs and other parenchymatous organs are frequent enough.

All these processes are, however, slow and irregular, without any tendency to a critical termination, and partake of the nature of mere congestion and infarction, with a disposition towards decomposition rather than to proper inflammatory plastic processes. Whilst alluding to certain periods of life as predisposing causes of cholosis, he takes occasion to notice icterus infantum, respecting which he seems to doubt what may be its true relation to biliary disease in general. He thinks it probable that many cases belong to primary cholosis, both from the symptoms during life and appearances detected after death. Some may be referred to secondary varieties of the disease, but there are not a few whose true circumstances and position remain to be determined by numerous and accurate observations. With respect to progress and duration, he defines the disease to be ("atypische") one *sui generis,* irregular in progress, undetermined as to limits, and of uncertain duration, which develops itself gradually or suddenly.

In some cases the symptoms disappear suddenly before they have become more than precursory, or they are so mild as scarcely to claim medical interference, or interrupt the patient's usual avocations.

The average duration of the disease is from four to six weeks. When prolonged beyond eight or ten weeks it passes into the secondary forms. It may, however, destroy life in eight or ten days. It is not the subject of proper critical efforts, for although a certain degree of alleviation sometimes follows deposits in the urine, general perspiration, discharges of blood, eruptions on the skin, and the restoration of suppressed secretions or excretions, yet Dr. H. does not consider them properly critical, because they do not appear at any regular period, are only accidental, and the disease often terminates favorably without them. Bilious stools frequently bring about a very decided improvement, but they have not always such a desirable result, nor is the time of their appearance determined; and, therefore, even they cannot be strictly called critical. The prognosis is generally favorable. Bilious dyscrasy sometimes, though not often, ends in other diseases; generally in bilious cachexia, with a disposition towards the reappearance of the original malady, or towards the development of its secondary forms. In such cases we have digestive derangement, chronic vomiting, irregular action or torpor of the liver, disposition towards the formation of gall-stones, shrinking and hardening of the liver, &c. It may also end in putrid fever; and if the brain has been much engaged, it may leave behind epilepsy, maniacal affections, or paralysis of the extremities.

When death does take place, Dr. Horaczek views it as the result of poisoning and paralysis of the cerebral nervous system, evidenced by the cephalic symptoms before enumerated, which follow the derangement or disturbance of the mutual relations between the nervous and vascular systems, and the subsequent softening of the organs chiefly implicated. Chemical analysis of the blood, and of the various secretions and excretions, is the most suitable, and, with the improvement of chemistry, will be the most certain method of diagnosticating cholosis.

We may thus determine whether cholosis, anemia, cachexia following the abuse of spirituous liquors, that which attends cancer, pyæmia, or purulence of the blood, phlebitis, &c., all of which are accompanied by more or less yellow discoloration of the surface, are also complicated with cholosis or not.

It is often difficult to distinguish primary cholosis from some of its secondary forms, dependent on disease of the liver, pancreas, pylorus, duodenum, pleura, &c., or on obstruction of the biliary ducts, feculent accumulations in the colon, or pregnancy. Disease of the hepatic parenchyma, attended with increase of volume, may, however, be detected by manual examination. There is, besides, in such cases emaciation, with long-continued dyspepsia and dropsical appearances; the yellowness, moreover, is earthy, the skin is dry, harsh, and faded, and the symptoms indicating the narcotising effects of the accumulated biliary constituents on the nervous system are not well pronounced.

It is difficult to diagnosticate jaundice from gall-stones, from primary cholosis, especially if it attack young persons under the influence of mental emotions, and with acute symptoms, such as vomiting, pain in the liver, &c. Gall-stones are, however, generally a disease of persons advanced in life; the pain they cause is colicky, attended by a sensation of burning heat, sometimes remitting, or coming on at intervals like the pains of labour, and it often ceases suddenly, or is relieved by remedial agents. There are also cramps of the abdominal muscles, and of the whole body; the skin is covered with cold sweat, the pulse is contracted, the head is not engaged, and inflammation of the liver or neighbouring parts often succeeds.

Polycholia cannot readily be confounded with primary cholosis, as it is attended by increased size and increased functional activity of the liver, together with an overflow of bile into the intestinal canal; as well as by an increased formation of biliary matter in the blood, and jaundice, consequent on its insufficient separation through the liver. To polycholia belong bilious fevers, and bilious pneumonia and pleurisy.

It remains to notice some of the chief therapeutical indications according to our author, as well as the means by which he proposes to work them out.

The causes of the disease, if still in activity, must if possible be removed, or their operation be neutralized.

The restoration of the disturbed relations between the functions of the vascular and nervous systems he lays down as another indication. In reference to which he remarks truly enough, that as the precise nature of this disturbance, not less than of the normal relations of these functions to each other, is and ever will be a mystery, we can only resort to such rational or well-devised empirical measures, as tend to restrain their activity when in excess, and to excite it when unduly depressed.

We are as yet acquainted with no agents calculated to neutralize the injurious effects of the biliary elements on the blood, except those found to be useful in other cachectic and dissolved conditions of that fluid; of which the best are the mineral and vegetable acids, and preparations containing chlorine. The most important indication is to eliminate the biliary admixture from the blood, by exciting the activity of the liver; for this purpose purgatives must be used, of which he prefers rhubarb and aloes in full doses. Calomel must not be given except in occasional and purgative doses, lest the plasticity of the blood be still further reduced; and for the same reason the author objects to saline aperients, which merely increase the intestinal secretions, except in those cases where it is desirable to make the intestinal canal the seat of a vicarious discharge of the biliary elements, in consequence of a total and persistent suspension of the functions of the liver.

If we are unable to restore the functional activity of the liver, the best substitute for it will be excitement of the kidneys by means of diuretics.

If symptoms of biliary oppression of the brain display themselves, the restoration of the hepatic functions must be attempted by strong emetics, drastic purgatives, dry cupping, stimulating applications to the abdomen, and stimulating enemata.

If the cerebral oppression be caused by hyperemia, or an inflammatory state of the brain, which is sometimes, though rarely, the case, we may resort to local bloodletting, cold applications to the head, sinapisms to the feet, or stimulating pediluvia, repeated doses of tartar emetic in decoction of tamarinds, and cooling acid drinks.

If the cerebral affection be purely nervous, besides external stimulants and revulsives applied to the head itself, as well as other parts of the body, the author resorts to the internal use of stimulants and tonics, enumerating in addition to those generally used in these countries, phosphorus and the flowers of the arnica, which last he prefers to all others, on account of its specific effects on the brain.

Pain in the liver, which is almost always purely nervous, he treats with external emollients, derivatives, and sedatives.

He subjoins a tolerably extended list of popular and superstitious remedies, to which additions, equally whimsical and disgusting, might be made from sources nearer home.

The book concludes with a detailed narrative of upwards of 90 cases. More than one third of the number terminated fatally, and the morbid appearances which they presented on post-mortem examination are also given, so that we are furnished with copious illustrations by which to test the doctrines and precepts inculcated by the author.

We here conclude our account of Dr. Horaczek's work, from which we have extracted largely. Although the author's pathological views are not always very definite, and are sometimes very open to criticism, we still think the book is well worthy of a careful perusal, as it supplies an important addition to our knowledge of a very obscure class of diseases, which it presents under aspects chemical, pathological, and even semeiological, in some respects both new and important; and induces us to look with considerable interest for the fulfilment of Dr. Horaczek's intimated design, of giving a complete view of the subject, by the publication of his researches on the other forms of bilious dyscrasy.

